# Early Cytokine Profiles in Critically Ill Patients with COVID-19 and Their Association with Mortality

**DOI:** 10.3390/metabo16040256

**Published:** 2026-04-11

**Authors:** Yenifer Gamarra-Morales, Jorge Molina-López, Juan Francisco Machado-Casas, Lourdes Herrera-Quintana, Héctor Vázquez-Lorente, José Miguel Pérez-Villares, Elena Planells

**Affiliations:** 1 Clinical Analysis Unit, Santa Ana Hospital, 18600 Motril, Spain; 2 Faculty of Education, Psychology and Sports Sciences, University of Huelva, 21007 Huelva, Spain; 3 Intensive Care Unit, Virgen de las Nieves Hospital, 18014 Granada, Spain; juanf.machado.sspa@juntadeandalucia.es (J.F.M.-C.); josem.perez.villares.sspa@juntadeandalucia.es (J.M.P.-V.); 4 Department of Physiology, School of Pharmacy, Institute of Nutrition and Food Technology “José Mataix”, University of Granada, 18071 Granada, Spain; lourdesherrera@ugr.es (L.H.-Q.); hectorvazquez@ugr.es (H.V.-L.); elenamp@ugr.es (E.P.)

**Keywords:** COVID-19, interleukin, mortality

## Abstract

Background/Objectives: The purpose of this study was to (i) determine the levels of interleukins in patients with COVID-19 admitted to the Intensive Care Unit (ICU) and (ii) evaluate their early dynamics, as well as (iii) assess their relationships with morbidity and mortality. Methods: This was a prospective analytical study of critically ill patients with COVID-19 who were monitored from admission to three days of stay in the ICU. Circulating levels of IL-1β, IL-2, IL-6, IL-7, IL-8, IL-10, and tumour necrosis factor-alpha (TNF-α) were measured. Cytokine levels were analysed in relation to clinical severity parameters and 28-day mortality. Results: A dynamic cytokine response was observed during the first 72 h, with a significant increase in TNF-α levels and a decrease in IL-10 and IL-1β. Non-survivors showed higher TNF-α levels than survivors. In the multivariable analysis adjusted for clinical severity, TNF-α remained independently associated with 28-day mortality, whereas other cytokines did not retain statistical significance. The overall predictive performance of cytokines was moderate. Conclusions: Early cytokine dynamics reflect the evolving inflammatory response in critically ill COVID-19 patients. TNF-α emerges as an independent predictor of mortality, supporting its role as a relevant biomarker of adverse outcomes. Although its predictive capacity is moderate, TNF-α may provide clinically meaningful information for risk stratification when integrated with established clinical and laboratory parameters.

## 1. Introduction

COVID-19 is an infectious disease that has spread worldwide, causing more than 6 million deaths, with a cumulative infection-to-mortality ratio of 0.4% [1,2]. The most severe manifestation of this disease results in patients being admitted to the ICU, where the mortality rate is almost 30% [3,4]. This high mortality is usually due to the combination of severe acute respiratory distress syndrome (ARDS), sepsis, and multiorgan failure. The cause of these alterations is usually the cytokine response [5,6] that triggers hyperinflammatory states. Thus, in COVID-19 disease, anti-inflammatory treatment [7] to curb the underlying alterations is more important than antiviral treatment.

ICU patients are highly heterogeneous due to hemodynamic instability and various medical interventions such as fluid therapy, renal replacement therapy, and mechanical ventilation. There is an increased risk of severe COVID-19 disease in male patients of advanced age with underlying diseases such as diabetes, chronic respiratory diseases, coronary artery disease, end-stage renal failure, and cirrhosis [8,9,10,11,12]. Progress is needed in identifying prognostic factors for COVID-19 severity in patients; among these, biomarkers related to poor clinical outcomes may be promising. These biomarkers include inflammatory markers that are often elevated on admission to the ICU, such as C-reactive protein (CRP), D-dimer (DD), and lactate dehydrogenase (LDH), along with lymphopenia and neutrophilia [13,14,15,16].

The inflammatory process that occurs in these patients is dynamic, characterised by the concurrent release of proinflammatory (TNF-α, IL-1ß, IL-12, IFN-γ, and IL-6) and anti-inflammatory (IL-1 receptor antagonist [IL-1 ra], transforming growth factor beta [TGF-ß], interleukins 4, 6, 10, 11, and 13) cytokines. The phase dominated by anti-inflammatory phenomena is associated with a decrease in nitric oxide production [17]. In pathological conditions, the inflammatory activity may not be counteracted by anti-inflammatory activity, or the anti-inflammatory activity can overwhelm the inflammatory effect, leaving the host at the mercy of the infection [18]. Blood levels of interleukins may be useful as biomarkers of the inflammatory status of these patients. As early as 1996, T. S. Blackwell observed that excess production of some of these proinflammatory cytokines (TNF-α, IL-1ß, IL-6, and IL-8) in the early phase of sepsis correlated with the development of multiorgan failure and increased mortality [19]. However, the magnitude, temporal evolution, and clinical relevance of these inflammatory alterations remain heterogeneous, and their role as independent prognostic markers is not clear.

Although numerous studies [20,21,22] have evaluated cytokine levels in COVID-19, most have relied on single time-point measurements, which may not adequately reflect the dynamic nature of the inflammatory response. The early phase of critical illness, particularly during the first days of ICU admission, represents a crucial window in which immune responses may evolve rapidly and influence clinical outcomes. A better understanding of the temporal profile of cytokines during this early phase may provide valuable insights into the pathophysiology of severe COVID-19 and help to identify inflammatory patterns associated with worse prognosis. In particular, determining whether specific cytokines are independently associated with mortality beyond established clinical severity scores remains clinically relevant.

Based on the aforementioned data, the overall objective of this study was to (i) determine interleukin levels (IL-1β, IL-2, IL-6, IL-7, IL-8, IL-10, and TNF-α) in patients with COVID-19 admitted to ICU and (ii) study their evolution over 72 h, as well as (iii) assess their relationships with morbidity and mortality. Specific objectives included the following: firstly, the comparison of interleukin levels with severity parameters such as Sequential Organ Failure Assessment (SOFA), Acute Physiology and Chronic Health Evaluation II (APACHE), days of mechanical ventilation, days of ICU stay, fraction of inspired oxygen (FiO_2_), and partial pressure of oxygen/fraction of inspired oxygen (PaO_2_/FiO_2_); secondly, the correlation of interleukin levels with inflammatory markers: fibrinogen, DD, CRP, and ferritin; and thirdly, the comparison of interleukin levels with mortality.

## 2. Materials and Methods

### 2.1. Study Design and Participants

This is a prospective, observational, analytical, follow-up study in critically ill patients with COVID-19. Patients admitted to the ICU with a diagnosis of COVID-19 from March to December 2020 at the Virgen de la Nieves Hospital in Granada (Spain) were recruited. The diagnosis of COVID-19 was confirmed by a positive real-time reverse transcriptase polymerase chain reaction (RT-PCR) test of nasal and throat swab samples and subsequent sequencing of SARS-CoV-2-specific RNA. The present study was conducted in accordance with the principles of the Declaration of Helsinki (last revised guidelines from 2013) [23], following the International Conference on Harmonization (ICH)/Good Clinical Practice (GCP) standards, and was approved by the Ethics Committee of the University of Granada (Ref. 149/CEIH/2016). Informed consent was collected from patients, their relatives, or close associates after being informed about the study protocol.

Patients selected for the study were those admitted to the ICU in critical condition, aged 18 years or older, who stayed at least 3 days, and had a positive PCR test for SARS-CoV-2 according to the Chinese Clinical Guideline for the Classification of COVID-19 [24]. Patients were considered critically ill when they had respiratory failure requiring mechanical ventilation, needed vasopressor treatment (shock), or had other complications with organ failure requiring monitoring or ICU treatment. Patients under 18 years of age, pregnant women, and those patients without a positive RT-PCR test for SARS-CoV-2 were excluded. Cytokine levels were measured at ICU admission (day 1) and after 72 h (day 3) in order to evaluate the early dynamics of the inflammatory response during the initial critical phase of the disease.

### 2.2. Data Collection

Patient information (age, sex, comorbidities, date of ICU onset and discharge, and COVID-19 CRP results) was collected in a database. Similarly, clinical parameters of the patients were collected on both the first and third day of ICU stay: days of ICU stay, days of mechanical ventilation, SOFA score [25] (calculated based on standard clinical and laboratory parameters including PaO_2_/FiO_2_ ratio, platelet count, bilirubin, mean arterial pressure or vasopressor requirement, Glasgow Coma Scale, and serum creatinine), APACHE II score [26] (measured only in the initial phase of the stay), and mortality. In addition, complementary parameters such as the mean arterial pressure, breathing rate, and other respiratory function variables such as FiO_2_ and PaO_2_/FiO_2_ were collected on both days.

Serum samples were obtained from the participants on the day of admission to ICU and on the third day in ICU and processed immediately. The following laboratory variables were analysed: (a) biochemical variables, including sodium, potassium, creatinine, alanine aminotransferase (ALT), aspartate aminotransferase (AST), gamma-glutamyl transferase (GGT), LDH, creatine kinase (CK); (b) haematological variables, including haemoglobin, haematocrit, leukocytes, percentage of lymphocytes, percentage of neutrophils, platelets, international normalized ratio (INR), and activated partial thromboplastin time (aPTT); and (c) inflammatory markers, including fibrinogen, DD, CRP, and ferritin. Biochemistry and immunochemistry parameters were determined using Alinity and Sysmex autoanalysers (Abbott Core Laboratory^®^, Lake Forest, IL, USA), employing enzymatic colorimetry and immunoassay. The remaining serum samples were frozen at −20 °C for the subsequent determination of interleukins.

### 2.3. Assessment of Interleukins

Multiplex panels (Ref: HCYTA-60K-20) were used to measure the following interleukins: IL-1β, IL-2, IL-6, IL-7, IL-8, IL-10, and TNF-α. The samples were processed on the LuminexR 200™ Systems (Merck Millipore, Merck KGaA, Darmstadt, Germany) using a multiplex bead-based immunoassay. The Luminex technology, based on flow cytometry, employed a set of beads with a different colour code assigned to each bead and conjugated with a specific reagent for each analyte of interest, allowing the simultaneous quantification of several analytes. The following reference values were considered: <13.6 pg/mL for IL-1β, <10 pg/mL for IL-2, <5.9 pg/mL for IL-6, <10 pg/mL for IL-7, <10 pg/mL for IL-8, <10 pg/mL for IL-10, and <12.4 pg/mL for TNF-α.

### 2.4. Statistical Analysis

All information was collected in a database, and statistical tests were processed using the statistical software SPSS version 21.0 (IBM SPSS, Armonk, New York, NY, USA). The GraphPad Prism 9 software (GraphPad Software, San Diego, CA, USA) was used to plot the graphs. A sample size calculation was performed to assess the difference between two means for a paired t-test, an alpha of 0.05, a power of 95%, and a mean effect size (0.05) (G*Power software, version 3.1.9.6, Kiel, Germany).

The assumption of normality was tested using the Kolmogorov–Smirnoff–Lilliefors test. Qualitative variables are presented as frequencies and percentages and quantitative variables as the median (interquartile range). The relationship of dichotomous qualitative variables was determined using the Chi-square test. For the association of quantitative variables with mortality and mechanical ventilation, we used the Mann–Whitney U test. Quantitative variables between day 1 and day 3 in the ICU were compared using the Wilcoxon signed-rank test for paired data to test the evolution of critically ill patients with COVID-19. Multiple comparisons were corrected using the Benjamini–Hochberg false discovery rate (FDR), and adjusted *p*-values (q-values) are reported. The association of interleukins with the rest of the quantitative variables was tested by applying Spearman’s correlation coefficient. The Cox proportional hazards model was used to estimate the risk of death adjusted for TNF-α levels categorised by median. Finally, a multivariable logistic regression analysis was performed, considering the SOFA score and cytokine levels with 28-day mortality. A *p*-value less than 0.05 was considered to be statistically significant.

## 3. Results

A total of 120 patients requiring admission to the ICU, staying there for at least 3 days, and meeting all the inclusion criteria were recruited. The sample size in this study was similar to that reported in previous research [27,28]. The study sample comprised 88 (73.3%) men and 32 (26.7%) women. Significant differences were observed in the frequency of men and women admitted to the ICU, with more men (χ^2^ = 22.1; *p* < 0.001). The mean age (p25th–p75th) was 63.0 (56.0–72.0), with an age range of 21 to 96 years.

Most patients had underlying diseases such as cardiovascular diseases, diabetes, hyperlipidaemia, and chronic obstructive pulmonary disease. The median (p25th–p75th) length of stay in the ICU was 14.0 (9.0–28.0) days, the number of days on mechanical ventilation was 9.0 (0–22.3), and the mortality rate at 28 days was 30.8%. A total of 85 (70.8%) patients required mechanical ventilation. Table 1 shows the clinical characteristics on the first and third days, and the fourth column shows the evolution after 3 days in the ICU. Significant decreases in the heart rate, breathing rate, and FiO_2_ were observed on the third day compared with the first day of admission.

The following interleukin results were obtained. Less than 1% of IL-1β and IL-2 levels were elevated. In total, 89.5% of the IL-6 levels were elevated on both the first and third days. Regarding IL-7, 20.8% of the levels were elevated on the first day, and 16.7% were elevated on the third day. In the case of IL-8, 95.8% of the levels were elevated on the first day and 98.3% on the third day. In total, 89.2% of the IL-10 levels were elevated on the first day and 81.6% on the third day. Finally, TNF-α was elevated in 62.5% of cases on the first day and 73.3% on the second day.

Table 2 shows the results of the interleukins and other laboratory variables measured on the first and third days in ICU and the evolution over that period using the Wilcoxon signed-rank test. The TNF-α levels showed a significant increase over time (*p* = 0.001; q = 0.001), whereas the IL-10 and IL-1β levels significantly decreased (*p* = 0.001 and *p* = 0.013, respectively; q ≤ 0.021). No significant changes were observed in the IL-2, IL-6, IL-7, and IL-8 levels. In addition, significant changes were observed in the biochemical and haematological variables and other inflammatory markers.

The mortality at 28 days was 30.8% (37 out of 120 patients). An association of interleukins with mortality at 28 days was performed, as shown in Table 3. TNF-α and IL-10 were increased significantly on the first and third days in those who died; that is, patients with higher levels were more likely to die. We found that IL-8 on the third day in ICU was also associated with mortality, because its values were higher in those who died than in the survivors.

**Figure 1:**
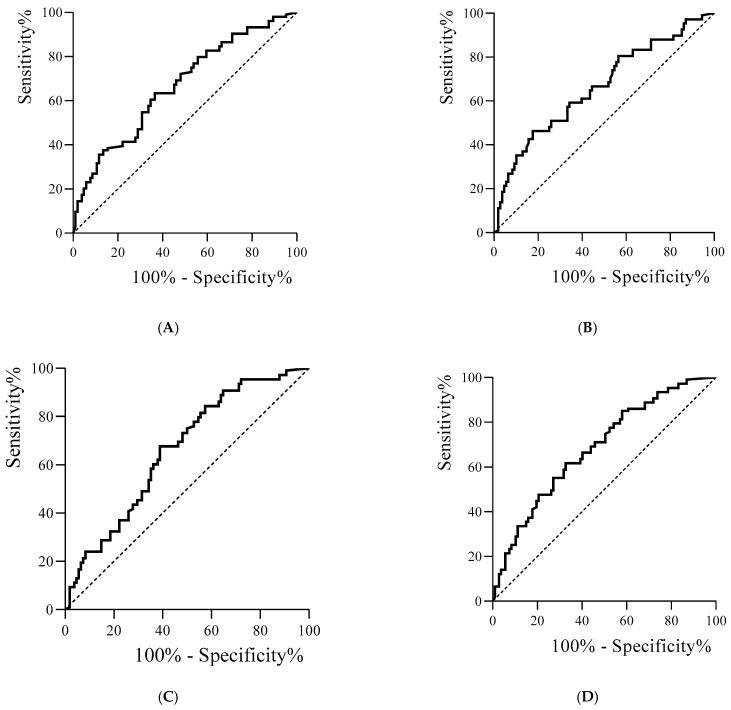
ROC curves for TNF-α on the first (**A**) and third (**B**) days and mortality. (**C**) ROC curve for IL-10 on the first day and mortality. (**D**) ROC curve for IL-10 on the third day and mortality.

Figure 1A,B shows the mortality receiver operating characteristic curve (ROC curve) with TNF-α. The areas under the curve were 0.678 (95% CI 0.567–0.789) for TNF-α on the first day (Figure 1A) and 0.676 (95% CI 0.561–0.790) for TNF-α on the third day (Figure 1B). Figure 1C shows the mortality ROC curve with IL-10 on the first day, which shows an area under the curve of 0.673 (95% CI 0.568–0.777). Figure 1D shows the mortality ROC curve with IL-10 on the third day, which shows an area under the curve of 0.697 (95% CI 0.593–0.801).

**Figure 2:**
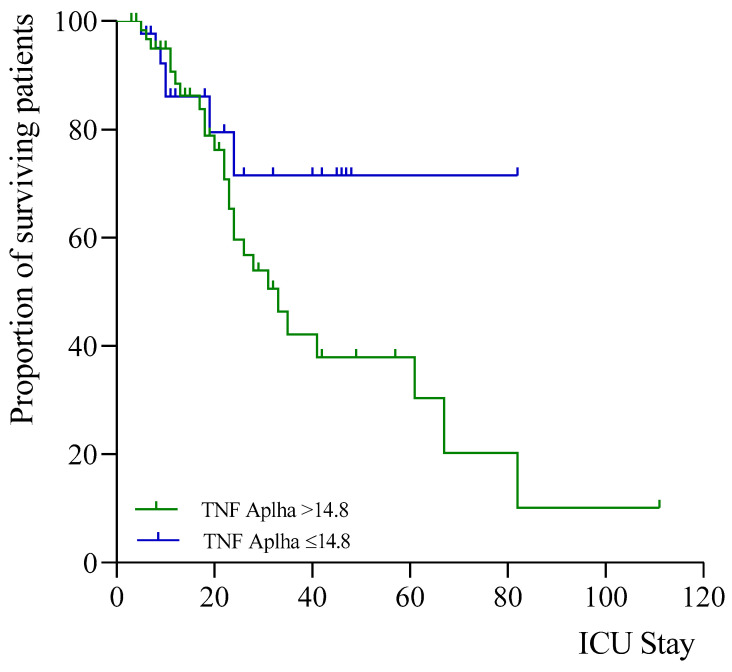
Survival curves of patients with COVID-19 based on TNF-α levels. The Cox regression model shows the overall survival for TNF-α levels categorised by the median. There was lower survival when the TNF-α levels were high (green, above cutoffs of 14.8 pg/mL) versus low (blue, below cutoffs). Each line indicates the predicted survival probability over follow-up time in ICU.

The median survival time was calculated for those patients who died. Survival was measured by whether the patients had high or low levels of TNF-α. A cut-off point was defined at 14.8 pg/mL (median of the values of our sample). The median survival was 63.0 days (95% CI 49.7–76.3) for low values of TNF-α and 45.1 days (95% CI 32.9–57.3) for high values of TNF-α. The association between the two groups with higher or lower levels of TNF-α and survival was calculated. A chi-square of 2.212 (*p* < 0.137) was obtained; hence, patients with higher levels of TNF-α died at a higher rate than those with lower levels of TNF-α. Figure 2 shows this difference in survival.

Interleukins were related to some variables involved in the severity of the disease, such as SOFA, APACHE, days of mechanical ventilation, days of stay in the ICU, FiO_2_, and PaO_2_/FiO_2_, as shown in Table 4. Most were positive correlations, whereby the higher the levels of interleukins, the higher the severity of the clinical variables.

Interleukins were also correlated with inflammation parameters such as fibrinogen, CRP, PCT, ferritin, and DD, as shown in Table 5. Most were positive correlations, whereby the higher the levels of interleukins, the higher the levels of inflammatory markers.

A multivariable logistic regression analysis including the SOFA score and the TNF-α and IL-10 levels was performed (Table 6). TNF-α remained independently associated with 28-day mortality, whereas neither the SOFA score nor the IL-10 were significantly associated with mortality.

## 4. Discussion

The purpose of the present study was (i) to determine the levels of interleukins in patients with COVID-19 admitted to the Intensive Care Unit (ICU) and (ii) to study their evolution over 72 h, as well as (iii) to assess their relationships with morbidity and mortality. First, the vast majority of interleukins were elevated in these patients on the first and third days. The serum levels of IL-1β and IL-2 were mostly within the reference values, and IL-7 was elevated in a very small number of patients. Therefore, we conclude that these would not be useful in the prognosis or follow-up of patients with COVID-19. As for IL-6, IL-8, IL-10, and TNF-α, they were elevated in the vast majority of patients. Therefore, they could be useful in the early prognosis and follow-up of the septic process in patients with COVID-19. Second, regarding ICU stay, decreases in IL-1β and IL-10, as well as an increase in TNF-α, were observed on the third day of ICU stay in critically ill COVID-19 patients. Third, regarding morbidity and mortality, TNF-α and IL-10 on the first day were higher in COVID-19 patients who died compared to those who survived. Moreover, IL-8 levels on the third day were also related to mortality, as patients with higher levels were more likely to die. The predictive performance observed in our study (AUC~0.65–0.70) suggests a moderate discriminatory ability. Therefore, cytokine levels should not be considered as standalone prognostic markers but, rather, as complementary biomarkers within a broader clinical and laboratory assessment. Importantly, TNF-α emerged as an independent predictor of mortality after adjustment for clinical severity, representing a key finding of this study. Additionally, we found an association between the interleukin levels and clinical and laboratory outcomes.

In our study, patients showed an improvement in clinical and laboratory outcomes during the first three days of stay in ICU. Specifically, a decrease in fibrinogen, CRP, and ferritin, along with an increase in DD, was observed on the third day, likely due to the supportive interventions following hospital protocols from ICU admission. The observed increase in TNF-α levels during the first 72 h suggests a persistent proinflammatory response in critically ill patients, whereas the decrease in IL-10 and IL-1β may reflect a dynamic modulation of the inflammatory and anti-inflammatory balance during the early phase of ICU admission. These findings highlight the dynamic nature of the inflammatory response in severe COVID-19, with a sustained increase in TNF-α potentially contributing to worse outcomes. This pattern in interleukins aligns with the model proposed by Siddiqi and Mehra [29]. These authors described a three-stage evolution of COVID-19 infection: stage 1 (mild), characterised by “early inflammation”; stage 2 (moderate), with pulmonary involvement, subdivided into IIa (without hypoxia) and IIb (with hypoxia); and stage 3 (severe), marked by “systemic hyperinflammation”. Our patients likely corresponded to this third stage. Here, inflammatory cytokines and biomarkers, including IL-2, IL-6, IL-7, granulocyte colony-stimulating factor, macrophage inflammatory protein 1-α, TNF-α, CRP, ferritin, and DD are elevated [30]. Additionally, a condition resembling secondary haemophagocytic lymphohistiocytosis may occur in patients in this advanced stage [7]. Tailored treatment in stage 3 relies on the use of immunomodulatory agents to reduce systemic inflammation and prevent overwhelming multi-organ dysfunction; the use of corticosteroids, along with the use of cytokine inhibitors such as tocilizumab (an IL-6 inhibitor) or anakinra (IL-1 receptor antagonist), would be warranted [7]. Overall, the prognosis in this critical phase would be poor, making early identification and timely therapeutic intervention crucial for improving outcomes. Consequently, these biomarkers may help to identify patients with a more pronounced inflammatory response, who could potentially benefit from closer monitoring or targeted immunomodulatory therapies. However, further studies are needed to determine whether cytokine-guided therapeutic strategies could improve clinical outcomes. In clinical practice, commonly used inflammatory biomarkers such as C-reactive protein, ferritin, and D-dimer are widely used for risk stratification in patients with COVID-19. Although cytokine measurements are not routinely available in most clinical settings, they may provide complementary information regarding the underlying inflammatory response. Hyperinflammatory responses have also been described in other SARS-CoV-2-related conditions, such as multisystem inflammatory syndrome in children (MIS-C) and adults (MIS-A). These syndromes are characterised by marked elevations of inflammatory cytokines and systemic immune dysregulation. MIS-C is a hyperinflammatory syndrome associated with SARS-CoV-2 that appears weeks after infection and is characterised by systemic inflammation and multi-organ involvement [31]. However, their clinical presentation and pathophysiology differ from the acute respiratory failure observed in critically ill COVID-19 patients.

IL-6, a biomarker that has been studied in inflammatory diseases, is a glycoprotein involved in inflammation whose release is induced by interleukin-1. In our study, we did not observe a relationship between IL-6 and mortality, which may be attributed to the use of tocilizumab in patients with the highest severity. The mechanism of action of this drug involves binding to both soluble and membrane-bound IL-6 receptors (IL-6Rs and IL-Rm) and inhibits IL-6-mediated signalling. IL-6 has been reported in a variety of conditions associated with inflammatory processes such as sepsis [32], neoplasms, autoimmune diseases, acquired immunodeficiency syndrome, alcoholic liver disease and infections, or transplant rejection [33,34,35,36,37]. Moreover, high IL-6 concentrations have also been found to be associated with both cardiovascular and all-cause mortality in the general elderly population [38]. In our study, IL-8 was associated with mortality on day 3, showing correlations with clinical outcomes of severity, with PaO_2_/FiO_2_ on both days, and with days of mechanical ventilation on day 3, as well as with biochemical severity-linked parameters, with DD on day 1 and with CRP on day 3. Additionally, several studies have suggested a direct relationship between IL-8 and severity in patients with sepsis [39,40,41] and COVID-19 [42]. Furthermore, a recent study [20] found that elevated serum levels of IL-6, IL-8, and TNF-α at admission were significantly associated with mortality (*p* < 0.001, *p* = 0.020, and *p* = 0.014, respectively).

Likewise, our study showed that IL-10 was related to mortality on both day 1 and day 3 of ICU stay. IL-10 levels were also correlated with clinical outcomes of disease severity, the number of days on mechanical ventilation (on both days), the ICU length of stay (on day 3), and FiO_2_ (on day 3). Furthermore, IL-10 was associated with biochemical markers of COVID-19 severity, such as CRP on both days and DD on the first day. In patients with neonatal sepsis in a hyperinflammatory state, the combination of IL-10, IL-8, and PCT has been shown to achieve early diagnosis of organ dysfunction [40]. A similar approach was observed using combined biomarkers such as IL-10, IL-17 and PCTs, demonstrating higher diagnostic accuracy compared to the individual markers [43].

In the present study, TNF-α showed a relationship with mortality on both days (first and third day of ICU stay) and with clinical variables of disease severity, including the number of days on mechanical ventilation (on both days) and the length of ICU stay (on the third day). TNF-α remained independently associated with mortality after adjustment for clinical severity as assessed by the SOFA score. This finding suggests that TNF-α may reflect a persistent inflammatory response that is not fully captured by conventional severity scores. In contrast, IL-10 did not retain independent significance, which may indicate that its elevation reflects a compensatory response rather than a direct association with mortality. TNF-α is essential for activating innate immunity against infectious agents, but dysregulation of TNF-α signalling can lead to severe complications. It is speculated that inflammation regulated by these cytokines leads to tissue destruction and pulmonary oedema through a mechanism of endothelial and epithelial cytoskeletal breakdown, thus allowing fluid influx during vasodilatation [44]. TNF-α recruits neutrophils in respiratory epithelia that are sensitised by the virus and release matrix metalloproteinase, which is strongly associated with irreversible pulmonary fibrosis in COVID-19 patients [45]. TNF-α levels have been found to be consistently higher in patients with severe COVID-19 [22] and in those suffering from comorbidities such as obesity, hypertension, and chronic heart failure [46,47,48]. TNF-α is a key proinflammatory cytokine involved in the activation of the amplification of systemic inflammation. Persistent elevation of TNF-α may reflect an uncontrolled inflammatory response contributing to tissue injury and organ dysfunction in critically ill patients. Conversely, IL-10 is an anti-inflammatory cytokine that may increase as a compensatory mechanism in response to excessive immune activation. Our study identifies TNF-α as a strong predictor of long-term survival, as shown in Figure 2. We did not find any other scientific studies that address this issue in detail. In our study, patients with TNF-α levels below 14.8 had an 80-day survival rate higher than 70%, whereas those with TNF-α levels above 14.8 had an 80-day survival rate below 10%.

Although the association between cytokine levels and COVID-19 severity has been widely reported, fewer studies have evaluated the short-term dynamic evolution of multiple cytokines during the first days in ICU and their relationship with clinical severity parameters. The present study provides additional insight into the temporal profile of inflammatory cytokines and their prognostic value in critically ill patients.

### Limitations

This study has several limitations that should be acknowledged. First, it was conducted in a single centre, which may limit the generalisability of the findings. Second, cytokine measurements were performed at only two time-points (ICU admission and 72 h later), which may not fully capture the dynamic evolution of the inflammatory response in critically ill patients. Third, the study population corresponds to patients admitted during the early phase of the COVID-19 pandemic, when therapeutic strategies differed from current clinical practice. Finally, although TNF-α was independently associated with mortality, the overall discriminatory performance of the cytokine levels was moderate, suggesting that these biomarkers should be interpreted in the context of other clinical and laboratory parameters rather than as standalone prognostic tools.

## 5. Conclusions

IL-8, IL-10, and TNF-α are useful for establishing a prognosis of morbidity and mortality in the early ICU admission of critically ill COVID-19 patients. Interleukin (IL-10, IL-1β) and TNF-α levels evolve in the first three days of ICU admission towards a state of increased hyperinflammation that is related to clinical and analytical variables. Notably, elevated TNF-α strongly predicted poor long-term survival, confirming its potential as a key prognostic biomarker. Our findings support the optimisation of critical care patient monitoring in COVID-19 through routine analysis of the comprehensive cytokine profile, enabling early risk stratification and improved clinical management of severe cases. This reinforces the value of multi-biomarker approaches in critical care settings.

## Figures and Tables

**Table 1:** Clinical characteristics and evolution after three days of critical patients with COVID-19.

n = 120	First Day Median (p25th–p75th)	Third Day Median (p25th–p75th)	*p*-Value
Age (years)	63.0 (56.0–72.0)		
ICU stay (days)	14.0 (9.0–28.0)		
MV (days)	9.0 (0.0–22.3)		
SOFA (score)	3.0 (3.0–4.0)	5.0 (3.0–7.0)	0.959
APACHE II (score)	13.0 (8.0–17.0)		
MAP (mmHg)	98.0 (81.8–110)	84.0 (75.0–95.8)	0.095
HR (bpm)	78.0 (65.0–89.0)	62.5 (52.3–80.0)	0.006 *
BR (rpm)	27.0 (22.0–30.0)	22.0 (19.0–24.0)	0.002 *
FiO_2_ (%)	0.85 (0.70–1.00)	0.60 (0.50–0.70)	0.001 **
PaO_2_/FiO_2_	149 (100–224)	200 (131–234)	0.646

Statistical significance: * *p* < 0.05; ** *p* < 0.01. MV: mechanical ventilation. SOFA: Sequential Organ Failure Assessments. APACHE II: Acute Physiology and Chronic Health Evaluation II. MAP: mean arterial pressure. HR: heart rate. BR: breathing rate. FiO_2_: fraction of inspired oxygen. PaO_2_/FiO_2_: partial pressure of oxygen/fraction of inspired oxygen.

**Table 2:** Evolution of interleukins and other biochemical and haematological parameters on the third day in the ICU for patients with COVID-19.

n = 120	First Day Median (p25th–p75th)	Third Day Median (p25th–p75th)	*p*-Value	q-Value (FDR)
**Biochemical variables**				
Sodium (mEq/L)	139 (137–141)	140 (137–144)	0.059	0.079
Potassium (mEq/L)	4.10 (3.70–4.30)	4.00 (3.70–4.40)	0.763	1.221
Creatinine (mg/dL)	0.81 (0.72–1.12)	0.74 (0.63–0.91)	0.001 **	0.004
ALT (U/L)	34.5 (23.0–47.5)	38.0 (25.0–62.8)	0.001 **	0.001
AST (U/L)	34.0 (23.0–46.5)	28.0 (20.0–42.5)	0.014 *	0.037
GGT (U/L)	60.0 (40.5–105.3)	95.5 (58.3–156.0)	0.001 **	0.001
LDH (U/L)	495 (414–621)	435 (352–510)	0.001 **	0.008
Creatine kinase (U/L)	76.0 (35.5–141.8)	39.0 (21.5–105.5)	0.007 *	0.014
**Haematological variables**				
Haemoglobin g/dL	13.5 (11.8–14.5)	12.6 (11.1–13.7)	0.001 **	0.008
Haematocrit (%)	38.8 (34.7–38.8)	36.8 (33.0–40.2)	0.001 **	0.003
Leukocytes * 10^3^/µL	9.67 (7.51–13.7)	8.80 (6.86–11.84)	0.013 *	0.026
Lymphocytes (%)	6.00 (3.68–9.03)	9.15 (5.40–13.83)	0.001 **	0.001
Neutrophils (%)	89.9 (86.1–92.8)	84.4 (77.6–89.9)	0.001 **	0.004
Platelets * 10^3^/µL	237 (197–295)	264 (204–343)	0.001 **	0.001
INR	1.08 (1.00–1.18)	1.06 (0.97–1.14)	0.088	0.141
APTT (s)	28.8 (26.9–32.2)	28.8 (26.8–31.1)	0.500	0.667
**Inflammatory markers**				
Fibrinogen (mg/dL)	678 (541–792)	573 (403–686)	0.001 **	0.003
DD (ng/mL)	980 (553–1633)	1400 (895–4550)	0.001 **	0.001
CRP (mg/L)	131.1 (52.9–187.9)	64.0 (23.5–122.6)	0.001 **	0.004
Ferritin (ng/mL)	1447 (720–2107)	1333 (740–2419)	0.028 *	0.028
IL-1β (pg/mL)	0.51 (0.01–0.95)	0.46(0.01–0.95)	0.013 *	0.021
IL-2 (pg/mL)	0.93 (0.29–1.63)	1.09 (0.30–1.57)	0.865	1.730
IL-6 (pg/mL)	44.0 (16.0–105.0)	47.0 (13.3–141.9)	0.109	0.079
IL-7 (pg/mL)	2.39 (0.08–7.50)	2.02 (0.04–7.05)	0.141	0.161
IL-8 (pg/mL)	53.7 (31.1–102.0)	69.3 (36.7–129.0)	0.073	0.097
IL-10 (pg/mL)	43.2 (18.6–81.8)	27.9 (12.2–49.8)	0.001 **	0.008
TNFα (pg/mL)	14.80 (8.98–23.30)	19.29 (11.01–31.41)	0.001 **	0.001

Statistical significance: * *p* < 0.05; ** *p* < 0.01. FDR: false discovery rate. ALT: alanine aminotransferase. AST: aspartate aminotransferase. GGT: gamma-glutamyl transferase. LDH: lactate dehydrogenase. INR: International Normalized Ratio. APTT: activated partial thromboplastin time. DD: D-dimer. CRP: C-reactive protein. IL-1β: Interleukin-1beta. IL-2: Interleukin-2. IL-6: Interleukin-6. IL-7: Interleukin-7. IL-8: Interleukin 8. IL-10: Interleukin-10. TNFα: tumour necrosis factor-alpha.

**Table 3:** Association of interleukins with 28-day mortality in patients with COVID-19.

n = 120	First Day			Third Day		
	Survivors Median (p25th–p75th)	Deceased Median (p25th–p75th)	*p*-Value	Survivors Median (p25th–p75th)	Deceased Median (p25th–p75th)	*p*-Value
IL-1β (pg/mL)	0.547 (0.01–1.26)	0.547 (0.269–0.871)	0.899	0.431 (0.010–0.976)	0.547 (0.19–1.04)	0.489
IL-2 (pg/mL)	1.15 (0.35–1.61)	1.31 (0.25–1.66)	0.947	1.20 (0.40–1.57)	1.32 (0.20–1.64)	0.912
IL-6 (pg/mL)	34.0 (15.7–87.6)	69.1 (15.9–203.0)	0.290	59.2 (15.8–172.7)	24.5 (10.1–66.0)	0.447
IL-7 (pg/mL)	2.40 (0.05–6.57)	2.30 (0.040–10.128)	0.861	1.29 (0.04–6.99)	3.90 (0.05–7.64)	0.185
IL-8 (pg/mL)	51.4 (31.6–86.6)	63.4 (31.0–162.7)	0.238	59.3 (26.8–107.7)	103.1 (44.0–144.5)	0.026 *
IL-10 (pg/mL)	34.1 (13.2–62.7)	52.6 (35.7–124.5)	0.004 *	19.7 (10.5–40.9)	43.3 (22.6–97.7)	0.001 **
TNFα (pg/mL)	13.2 (6–20.4)	19.0 (12.9–35.6)	0.003 *	16.1 (10.7–28.0)	25.5 (16.8–60.5)	0.004 *

Statistical significance: * *p* < 0.05; ** *p* < 0.01. IL-1β: Interleukin-1beta. IL-2: Interleukin-2. IL-6: Interleukin-6. IL-7: Interleukin-7. IL-8: Interleukin 8. IL-10: Interleukin-10. TNFα: tumour necrosis factor-alpha.

**Table 4:** Association between interleukins and clinical parameters of ICU patient severity.

n = 120		SOFA	APACHE II	MVD	ICU Stay	FiO_2_	PaFi
First day	IL-1β (pg/mL)	−0.152	0.028	−0.164	−0.220 *	−0.052	0.057
	IL-2 (pg/mL)	0.004	−0.079	−0.123	−0.185	0.088	−0.161
	IL-6 (pg/mL)	0.280	0.127	−0.164	−0.200	0.262	0.058
	IL-7 (pg/mL)	−0.333 *	−0.011	−0.082	−0.087	0.164	−0.133
	IL-8 (pg/mL)	0.100	0.085	0.085	−0.050	0.032	−0.415 *
	IL-10 (pg/mL)	0.198	0.201	0.198 *	0.072	0.028	0.070
	TNFα (pg/mL)	0.178	0.171	0.222 *	0.122	−0.172	−0.089
Third day	IL-1β (pg/mL)	−0.013		−0.083	−0.136	0.017	−0.205
	IL-2 (pg/mL)	0.103		0.070	−0.132	0.002	−0.163
	IL-6 (pg/mL)	−0.074		−0.233	−0.206	0.171	0.097
	IL-7 (pg/mL)	0.626		−0.032	−0.009	−0.013	−0.032
	IL-8 (pg/mL)	0.256		0.276 *	0.147	0.034	−0.336 *
	IL-10 (pg/mL)	0.305		0.377 **	0.289 *	−0.219 *	−0.114
	TNFα (pg/mL)	0.274		0.276 *	0.214 *	−0.062	−0.114

Statistical significance: * *p* < 0.05; ** *p* < 0.01. SOFA: Sequential Organ Failure Assessments. APACHE II: Acute Physiology and Chronic Health Evaluation II. MVD: mechanical ventilation days. ICU: Intensive Care Unit. IL-1β: Interleukin-1beta. IL-2: Interleukin-2. IL-6: Interleukin-6. IL-7: Interleukin-7. IL-8: Interleukin 8. IL-10: Interleukin-10. TNFα: tumour necrosis factor-alpha.

**Table 5:** Correlations between interleukins and inflammatory markers.

n = 120		Fibrinogen	DD	CRP	Ferritin
First day	IL-1β (pg/mL)	0.005	−0.021	−0.040	0.007
	IL-2 (pg/mL)	0.059	−0.045	0.072	0.082
	IL-6 (pg/mL)	−0.003	0.165	0.138	0.083
	IL-7 (pg/mL)	−0.030	−0.006	−0.082	−0.186
	IL-8 (pg/mL)	0.044	0.202 *	0.137	−0.093
	IL-10 (pg/mL)	0.007	0.189 *	0.281 *	0.023
	TNFα (pg/mL)	0.139	0.130	0.104	0.053
Third day	IL-1β (pg/mL)	0.162	−0.106	0.069	0.053
	IL-2 (pg/mL)	−0.001	−0.065	−0.022	0.055
	IL-6 (pg/mL)	−0.029	0.593 *	0.261	0.084
	IL-7 (pg/mL)	0.024	−0.037	0.059	−0.106
	IL-8 (pg/mL)	0.191	0.010	0.334 **	0.007
	IL-10 (pg/mL)	0.148	0.101	0.355 **	0.059
	TNFα (pg/mL)	0.051	0.032	0.121	0.079

Statistical significance: * *p* < 0.05; ** *p* < 0.01. DD: D-dimer. CRP: C-reactive protein. IL-1β: Interleukin-1beta. IL-2: Interleukin-2. IL-6: Interleukin-6. IL-7: Interleukin-7. IL-8: Interleukin-8. IL-10: Interleukin-10. TNFα: tumour necrosis factor-alpha.

**Table 6:** Multivariable logistic regression analysis for 28-day mortality.

n = 120	OR	IC 95%	*p*-Value
SOFA	1.203	0.890–1.625	0.229
IL-10	0.997	0.987–1.008	0.624
TNF-α	1.059	1.004–1.117	0.034 *

Statistical significance: * *p* < 0.05. SOFA: Sequential Organ Failure Assessments. IL-10: Interleukin-10. TNFα: tumour necrosis factor-alpha.

## Data Availability

The data that support the findings of this study are available from the corresponding author, [Y.G.-M. and/or J.M.-L.], upon reasonable request.

## Abbreviations

The following abbreviations are used in this manuscript:

ALT	Alanine aminotransferase
APACHE II	Acute Physiology and Chronic Health Evaluation II
APTT/aPTT	Activated partial thromboplastin time
ARDS	Acute respiratory distress syndrome
AST	Aspartate aminotransferase
BR	Breathing rate
CI	Confidence interval
CK	Creatine kinase
COVID-19	Coronavirus Disease 2019
CRP	C-reactive protein
DD	D-dimer
ELISA	Enzyme-linked immunosorbent assay
FDR	False discovery rate
FiO_2_	Fraction of inspired oxygen
GCP	Good Clinical Practice
GGT	Gamma-glutamyl transferase
HR	Heart rate
ICH	International Conference on Harmonization
ICU	Intensive Care Unit
IFN-γ	Interferon-gamma
IL-1 ra	IL-1 receptor antagonist
IL-1β	Interleukin-1beta
IL-2	Interleukin-2
IL-4	Interleukin-4
IL-6	Interleukin-6
IL-6Rm	Membrane-bound IL-6 receptors
IL-6Rs	Soluble IL-6 receptors
IL-7	Interleukin-7
IL-8	Interleukin-8
IL-10	Interleukin-10
IL-11	Interleukin-11
IL-12	Interleukin-12
IL-13	Interleukin-13
IL-17	Interleukin-17
INR	International normalized ratio
LDH	Lactate dehydrogenase
MAP	Mean arterial pressure
MIS-A	Multisystem inflammatory syndrome in adults
MIS-C	Multisystem inflammatory syndrome in children
MV/MVD	Mechanical ventilation / Mechanical ventilation days
PaFi	PaO_2_/FiO_2_ ratio
PaO_2_/FiO_2_	Partial pressure of oxygen/Fraction of inspired oxygen
PCT	Procalcitonin
ROC	Receiver operating characteristic
RT-PCR	Real-time reverse transcriptase polymerase chain reaction
SARS-CoV-2	Severe acute respiratory syndrome coronavirus 2
SD	Standard deviation
SOFA	Sequential Organ Failure Assessment
TGF-ß	Transforming growth factor beta
TNF-α	Tumour necrosis factor-alpha
